# Tubulointerstitial Nephritis and Uveitis Syndrome: Case Series and Literature Review

**DOI:** 10.1155/2021/1812271

**Published:** 2021-06-02

**Authors:** Beatriz Oliveira Lopes, Margarida Sena Brízido, Ana Cortesão Costa, Mário Raimundo, Margarida Maria Miranda, Susana Morais Pina

**Affiliations:** ^1^Department of Ophthalmology, Beatriz Ângelo Hospital, Loures, Portugal; ^2^Department of Nephrology, Beatriz Ângelo Hospital, Loures, Portugal

## Abstract

Tubulointerstitial nephritis and uveitis syndrome (TINU) is a rare oculorenal inflammatory entity with a probable autoimmune etiology. Interstitial nephritis may be asymptomatic and usually has a benign course with spontaneous resolution. Uveitis, instead, is classically anterior, bilateral, and nongranulomatous, but it can be unilateral and presents as posterior uveitis or panuveitis, sometimes with a chronic or recurrent evolution. The frequent time lag of ocular and renal manifestations makes this diagnosis particularly challenging. The authors describe four cases of this rare entity, two with tubulointerstitial nephritis preceding ocular manifestations and the remaining, instead, with uveitis preceding renal involvement. The therapeutic approach included systemic corticosteroids in all cases. The addition of immunosuppressive therapy was required in three patients to achieve uveitis control. TINU is probably an underrecognized entity and should always be considered in the differential diagnosis of a chronic or recurrent idiopathic uveitis, especially in young patients who may have mild and asymptomatic renal disease.

## 1. Introduction

Tubulointerstitial nephritis and uveitis (TINU) syndrome is a rare inflammatory autoimmune disease [1, 2], described for the first time by Dobrin et al. in 1975 [3]. It is defined by the association of uveitis and acute tubulointerstitial nephritis (TIN) and requires exclusion of infection or other potentially associated systemic diseases [4, 5]. The syndrome is an important cause of acute kidney injury, responsible for 5% of the cases of acute TIN [4, 6]. In contrast, only, 0.1-2% of uveitis had been related to TINUS [1, 7, 8]. The subclinical nature of renal manifestations [5, 7] and the possible interlude with ophthalmic presentation make this diagnosis a challenge in clinical practice.

Our purpose is to alert for the existence of this clinical entity and highlight for the importance of screening for renal disease in patients with chronic or recurrent nongranulomatous uveitis of unclear etiology reviewing a series of four clinical cases of TINU.

## 2. Clinical Cases

### 2.1. Patient 1

A 49-year-old female presented to the emergency room with lumbar pain, transitory dark urine, asthenia, anorexia, weight loss, and leg and periorbital edema (Table 1). She referred chronic use of a proton pump inhibitor and had been consuming a nonsteroidal anti-inflammatory drug (NSAID) for the previous month.

Laboratory evaluation showed elevation of the erythrocyte sedimentation rate (ESR) 140 mm/first hour and C-reactive protein (CRP) 7.75 mg/dL, normocytic and normochromic anemia (Hb 10.1 g/dL), and increased serum creatinine (3.96 mg/dL) and urea (103 mg/dL). Urinalysis showed hematuria and proteinuria (1044 mg/24 h) without cellular casts. Additional investigations were unremarkable. She underwent a kidney biopsy for declining kidney function despite intravenous hydration and discontinuation of nephrotoxins that showed, on light microscopy, normal glomeruli and diffuse mononuclear cell interstitial infiltrates, consistent with acute TIN, which was initially deemed pharmacologic.

**Figure 1:**
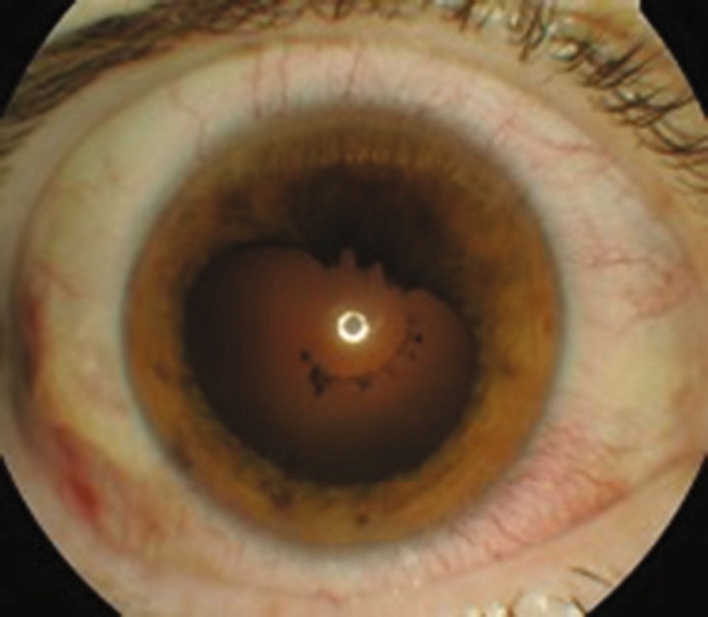
Photograph of the anterior segment of the right eye after pharmacological mydriases, showing posterior synechia between 12 and 2 h. Deposition of the iris pigment in the anterior capsule due to posterior synechia (patient 1).

She started systemic corticosteroids (oral prednisolone 1 mg/kg/day) with clinical improvement and full renal function recovery in 6 months. However, at 8 months after the initial presentation, while on the final phase of steroid tapering (prednisolone 2.5 mg every other day), she presented with ciliar hyperaemia and pain in the right eye (Table 1) and unilateral anterior nongranulomatous uveitis was detected (Figure 1).

Based on the presence of uveitis and TIN, the diagnosis of TINU syndrome was confirmed. Further clinical investigation was made to rule out the main infection and inflammatory diseases considered for differential diagnosis, including hepatitis B and C, *Toxoplasma gondii*, *Mycobacterium tuberculosis*, Epstein-Barr, syphilis, cytomegalovirus, and brucellosis infections, sarcoidosis, and Behcet's disease. Examination for human leukocyte antigen (HLA) alleles showed HLADQ B1∗02∗06 HLADR B1∗02∗06 positivity. Systemic corticosteroids were optimized to 0.5 mg/kg/day, and azathioprine 2 mg/kg/day was associated, beyond topical corticosteroids, with achieved uveitis control (Table 1). Six years after being diagnosed, the patient is clinically stable under immunosuppressive therapy with prednisolone 5 mg/day and azathioprine 1 mg/kg/day.

### 2.2. Patient 2

A 54-year-old female presented to the emergency room with urinary frequency, asthenia, anorexia and progressive weight loss (Table 1). She had diabetes without known microvascular complications, dyslipidaemia, hypertension, and sporadic use of NSAIDs.

**Figure 2:**
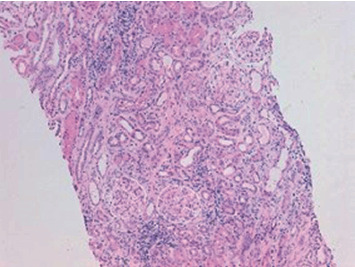
Pathological specimen obtained by renal biopsy (×100). Hematoxylin-eosin. Diffuse infiltration of inflammatory cells, mainly consisting of lymphocytic infiltrate, interstitial fibrosis, and tubular atrophy. The glomeruli are normal (patient 2).

Analysis showed normocytic normochromic anemia (Hb 6 g/dL), metabolic acidosis, elevation of ESR (88 mm/first hour) and CRP (3.87 mg/dL), increased serum urea (171 mg/dL) and creatinine (7.7 mg/dL), nonnephrotic proteinuria (484 mg/24 h), haematuria, and leukocyturia without cellular casts. Additional investigations, namely, for autoimmune diseases and multiple myeloma, were negative, and a kidney biopsy was performed which was consistent with acute TIN (Figure 2).

She started oral steroids (1 mg/kg/day) with progressive dose tapering which resulted in clinical and kidney function improvement. One year later, treatment was discontinued motivated by apparent clinical control. However, two weeks after treatment interruption, she presented with ocular pain in the right eye (Table 1). Unilateral nongranulomatous anterior and intermediate uveitis was detected, and TINU syndrome was suspected.

Further evaluation included exclusion of infections and other autoimmune diseases. Evaluation for HLA genotypes was positive for HLADQ B1∗03∗05. Beyond topical corticosteroids and mydriatics, systemic corticosteroids (1 mg/kg/day) were reintroduced with slow progressive withdrawal, lasting two years, on account of recurrent uveitis (Table 1). Currently, the patient remains clinically stable with no new recurrences and no need for corticosteroid therapy for the last 4 years.

### 2.3. Patient 3

A previously healthy 32-year-old female presented to the emergency room with hyperaemia and bilateral ocular pain for a week. Ophthalmology observation revealed bilateral anterior nongranulomatous uveitis and anterior vitritis. She was prescribed topical corticosteroids, mydriatics, and NSAIDs with transitory clinical improvement. A few weeks later, uveitis recurred and she also reported asthenia, arthralgia, and myalgia (Table 1).

Investigation revealed hypergammaglobulinemia (IgG 1900 mg/dL), creatinine (1.58 mg/dL) and urea (60 mg/dL) elevation, and raised levels of ESR (61 mm/hour), CRP (2.3 mg/dL), and urine *β*2-microglobulin (661 mg/dL). Urinalysis showed proteinuria and hematuria. The examination for HLA genotypes was positive for HLADR B1∗01. Infections and other autoimmune diseases were ruled out.

Based on clinical criteria, TIN was considered and diagnosis of TINU syndrome was assumed. The patient started systemic corticosteroids (0.5 mg/kg/day) with a dose tapering schedule with progressive ocular resolution. Six months later, she started azathioprine (2 mg/kg/day) with subsequent need to increase the dose to 2.5 mg/kg/day on account of uveitis recurrence (Table 1). Currently, the patient is clinically stable after 2 years on immunosuppressive therapy.

### 2.4. Patient 4

A healthy 16-year-old male came to the emergency room with ocular pain and redness associated with bilateral diminished visual acuity for five days. He presented anterior bilateral nongranulomatous uveitis with posterior synechiae, vitritis, and diffuse scattered soft white subretinal dots compatible with multifocal choroiditis. Considering a bilateral panuveitis, an etiologic study was started and he was prescribed mydriatics and topical steroids (Table 1).

Despite slight ophthalmic improvement, 2 weeks later, he presented with fever, myalgias, anorexia and weight loss. Laboratory evaluation showed hypergammaglobulinemia (IgG 1620 mg/dL), creatinine elevation (1.5 mg/dL), nonnephrotic proteinuria (82 mg/24 h), haematuria, leukocyturia, and elevated urine *β*2-microglobulin (1120 mg/dL). The main infections and inflammatory diseases were ruled out. Kidney injury was attributed to TIN, and diagnosis of TINU syndrome was established. He started prednisolone (1 mg/kg/day) with progressive withdrawal and methotrexate (25 mg/week) which resulted in uveitis control and kidney function recovery within 4 months (Table 1). After two years of clinical stabilization, it was possible to discontinue immunosuppressive therapy with no new recurrences.

## 3. Discussion

This paper reports four cases of TINU syndrome, a rare entity with higher incidence in young females with a median age at presentation of 15 years [2, 5, 7]. Recently, cases in adults and male patients with a wider range of presentations have been described [2, 5, 9], as is evident in our case series.

The pathogenesis of the syndrome remains unclear, and it is probably multifactorial [2, 4]. An autoimmune process involving cellular and humoral immunity, possible against a common ocular and kidney antigen target, may have an important role [5, 7, 10]. A loss of T-cell tolerance may be suggested by the presence of some high-risk human leukocyte antigen genotypes (HLA-A2, HLA-A24, HLADQA1∗01, HLADQB1∗05 and HLADRB1∗01, and HLADRB1 ∗0102) in TINU syndrome patients, as described in the literature [1, 2, 11]. In our series, only patient 2 had one of this risk HLA alleles; however, the haplotypes identified in the other patients may contribute to the pathogenesis of the disease as well. Published papers also described the association with drugs, including some antibiotics and NSAIDs, and infections predominantly by *Toxoplasma gondii*, *Mycobacterium tuberculosis*, Epstein-Barr, and varicella-zoster viruses [2, 4, 8], risk factors involved in TINUS etiology [8]. Sporadic use of NSAIDs was documented in patients 1 and 2.

The diagnosis of this entity requires the presence of TIN and typical uveitis with no other systemic or infectious disease [8, 12]. Although kidney biopsy is considered the classic and definitive diagnostic test [1, 4, 7], TIN can be inferred by the presence of complete clinical criteria published by Mandeville et al. [2] and also found in our patients: abnormal kidney function, urinalysis alterations (elevated *β*2-microglobulin, low-grade proteinuria, haematuria, glycosuria, leukocyturia, urinary eosinophils, or white blood cell casts), and systemic manifestations lasting at least for two weeks, with the last one characterized by any combination of signs and symptoms such as fever, fatigue, weight loss, and laboratory abnormalities, including anemia, abnormal liver function, eosinophilia, or elevated ESR.

In the first two patients, since TIN was the first manifestation, a kidney biopsy was promptly made, and after the development of uveitis, the diagnosis was confirmed. In the last 2 patients, a kidney biopsy was not performed as kidney involvement was mild, and the procedure was not considered of clinical benefit. Early kidney function evaluation and urinalysis were imperative in these cases, as well as *β*2-microglobulin urinary levels. *β*2-Microglobulin is a low molecular weight protein which is freely filtered in the glomerulus and, in the normal condition, reabsorbed in the proximal tubule. This resorptive process is impaired in TIN, making *β*2-microglobulin a sensitive and specific noninvasive diagnostic test in the appropriate clinical setting [1, 4, 12].

Differential diagnosis should comprise the most common causes responsible for the coexistence of uveitis and TIN, such as sarcoidosis, tuberculosis, Sjogren's syndrome, lupus, Wegener's granulomatosis, and Behcet's disease [1, 5, 8], which always need to be ruled out.

Uveitis is typically bilateral, nongranulomatous, and anterior [1, 7, 13]. However, involvement of vitreous or choroid may also be present [4, 7, 8], as observed in patients 2, 3, and 4. In 65% of cases, uveitis takes place after the TIN [2, 12] and can occur until 12 months after its onset [12]. Less commonly (21%), it can precede nephritis, usually less than 2 months before [2]. In only 15% of patients, uveitis occurs simultaneously with TIN, which makes the diagnosis particularly challenging [2, 12].

Uveitis may be chronic or recurrent in 50% of the cases [1, 2, 7]. Prolonged treatment with immunosuppressive therapy is usually required. Kidney involvement, instead, is often subclinical and generally resolves spontaneously [2, 4, 5], although the development of chronic kidney disease may occur in 11% of patients [2, 5, 14], mostly related to treatment delay. In all presented cases, TIN was transitory with good response to systemic corticosteroids.

The best visual and renal prognosis depends on the early diagnosis and timely treatment of this medical entity. Therefore, it is essential to raise awareness of the importance of screening for renal disease in patients with chronic or recurrent uveitis of unclear cause.

## Figures and Tables

**Table 1:** Description of the presented cases.

	Case 1	Case 2	Case 3	Case 4
Sex	Female	Female	Female	Male
Age (years)	49	54	32	16
Clinical presentation	Lumbar pain, dark urine, asthenia, anorexia, weight loss, leg and periorbital edema, unilateral anterior uveitis	Urinary frequency, asthenia, anorexia, weight loss, unilateral anterior and intermediate uveitis	Bilateral anterior and intermediate uveitis, asthenia, arthralgia, and myalgia	Bilateral panuveitis, fever, myalgias, anorexia, and weight loss
First ocular symptoms			+	+
First renal symptoms	+	+		
Clinical diagnosis			+	+
Histological diagnosis	+	+		
Ocular treatment	Topical corticosteroid, mydriatic	Topical corticosteroid, mydriatic	Topical corticosteroid, mydriatic	Topical corticosteroid, mydriatic
Systemic treatment	Oral prednisolone (1 mg/kg/day) with progressive withdrawal, azathioprine (2 mg/kg/day)	Oral prednisolone (1 mg/kg/day) with progressive withdrawal	Oral prednisolone (1 mg/kg/day) with progressive withdrawal, azathioprine (2 mg/kg/day)	Oral prednisolone (1 mg/kg/day) with progressive withdrawal, methotrexate (25 mg/week)
Follow-up	Uveitis recurrence: once	Uveitis recurrence: once	Uveitis recurrence: twice	No recurrence

## Data Availability

Data are available within the article.
